# Do Hormone Levels Influence Bullying during Childhood and Adolescence? A Systematic Review of the Literature

**DOI:** 10.3390/children11020241

**Published:** 2024-02-14

**Authors:** Izaro Babarro, Ane Arregi, Ainara Andiarena, Nerea Lertxundi, Oscar Vegas, Jesus Ibarluzea

**Affiliations:** 1 Faculty of Medicine and Nursing, University of the Basque Country (UPV/EHU), 20014 Donostia-San Sebastian, Spain; izaro.babarro@ehu.eus; 2 Group of Environmental Epidemiology and Child Development, Biogipuzkoa Health Research Institute, 20014 Donostia-San Sebastian, Spain; 3 Faculty of Psychology, University of the Basque Country (UPV/EHU), 20018 Donostia-San Sebastian, Spain; 4 Spanish Consortium for Research on Epidemiology and Public Health (CIBERESP), Instituto de Salud Carlos III, 28029 Madrid, Spain; 5 Sub-Directorate for Public Health and Addictions of Gipuzkoa, Ministry of Health of the Basque Government, 20013 Donostia-San Sebastian, Spain

**Keywords:** bullying, testosterone, cortisol, children and adolescents

## Abstract

(1) Background: Bullying is one of the most common forms of aggressive behavior during childhood and adolescence. Some decades ago, researchers began exploring the basis of peer victimization from a biological perspective. Specifically, the Hypothalamic-Pituitary-Adrenal (HPA) and Hypothalamic-Pituitary-Gonadal (HPG) axes have been studied in relation to status-relevant behaviors, such as bullying. (2) Methods: We conducted a systematic review following the PRISMA guide and registered the review protocol at PROSPERO (CRD42023494738). We searched for relevant studies in PubMed, Psycinfo, Scopus, and Web of Science, and assessed them using the Robins E-tool. (3) Results: Our search yielded 152 studies, of which 33 were included in the review. These studies explored the association between testosterone and cortisol levels with bullying behavior, finding diverse results. Most of the studies were rated as having a low risk of bias. (4) Conclusions: This study not only enhances our understanding of bullying, but also provides guidance for the development of prevention and management programs for it. In the future, researchers should continue exploring the joint effects of different hormones on the HPA and HPG axis, using a broader set of biomarkers.

## 1. Introduction

Bullying is a common form of aggressive behavior during childhood and adolescence, and it has been identified as one of the main sources of stress during these periods [1,2]. This behavior is defined as a type of aggressive behavior that occurs in the school environment and it is characterized by three main aspects: intentionality, repeatability, and power imbalance [3]. Several studies have examined this phenomenon and concluded that there are diverse types of bullying, including physical, verbal, social, psychological, or cyberbullying [4,5].

Recent evidence indicates that one in three students are involved in bullying worldwide [1]. Some years ago, The World Health Organization (WHO) estimated that between 2 and 32% of students were victims of bullying, while between 1 and 36% were bullies [6]. Typically, bullying starts between the ages of 7 and 8 years and reaches its peak between 11 and 14 years [7], after which it decreases and remains stable [8,9,10]. Gender differences have also been observed, with boys being more likely to be involved as victims, bullies, or bullies/victims [8,11,12]. However, it has also been concluded that there are differences in bullying prevalence based on gender. For instance, girls are more likely to be defenders [3]. In addition, a recent systematic review carried out in 2021, found that the prevalence varied by gender depending on the type of bullying. They concluded that although boys were generally more likely to be victims of bullying, girls were more involved in social bullying [12].

Bullying behavior can have negative impact on children’s and adolescents´physical and psychosocial lives. Due to its consequences and high prevalence, bullying is now considered a public health problem [13,14,15,16]. Some decades ago, researchers began studying the physiology behind peer victimization from a biological perspective, expanding their efforts beyond previous behavioral psychological and social models. Studies showed that epigenetic alterations, inflammatory markers, and neuroendocrine factors were associated with bullying behavior [4,17]. Hormone levels have also been studied in relation to bullying, as some hormones may affect behavior. Additionally, certain behaviors can also alter hormone levels.

The neuroendocrine system comprises the hypothalamus and pituitary gland, which are responsible for controlling the main hormonal axes in the body. Of particular interest in relation to status-relevant behaviors like bullying are the Hypothalamic-Pituitary-Adrenal (HPA) and Hypothalamic-Pituitary-Gonadal (HPG) axes [18]. According to the dual hormone hypothesis, basal cortisol and testosterone levels, which are products of the HPA and HPG axes, respectively, affect behavioral systems implicated in dominance and aggression. Several studies have described an association between high testosterone levels and high aggression when cortisol levels are low [19]. However, it is still unclear what happens during childhood and adolescence, which are crucial developmental stages for brain and cognitive maturation.

On the one hand, the HPA axis is responsible for the body’s stress response with cortisol as the final product. The association between cortisol levels and bullying behavior can be bidirectional. First, considering bullying as one of the main stressful events during childhood and adolescence, it can be thought that it influences HPA activity. According to the dual hormone hypothesis, we would also expect an association between testosterone and cortisol, resulting in aggressive behavior. Recently, a systematic review explored the association between cortisol levels and bullying behavior. They concluded that bullying was consistently associated mainly with blunted cortisol reactivity and diurnal cortisol slope. However, although being a statistically significant association, the direction of this relationship is still unclear [20].

On the other hand, the HPG axis, which controls the reproductive system, has been widely studied in relation to aggression. The central nervous system (CNS) is affected by hormones in the body, especially at two developmental stages: the prenatal and pubertal periods [21,22,23]. During this developmental stage, sex hormones can influence various brain structures [23], organizing and activating neuroendocrine circuits that control behavior [24,25]. Although some review studies and meta-analyses have found a statistically positive significant association between prenatal and pubertal sexual hormones and aggressive behavior [26,27,28], only a few studies have analyzed the association between sex hormone levels and bullying behavior, they have found mixed results [29,30,31].

Overall, it is still unclear in which direction cortisol is associated with bullying, and few studies have explored testosterone´s role in bullying. Therefore, this study aims to examine the relationship between sex hormones and cortisol levels with bullying behavior. The study will explore not only the direct association between cortisol and bullying but also the moderating role of cortisol.

## 2. Materials and Methods

The method used in this systematic review is described in a protocol registered on PROSPERO (reference CRD42023494738 available from https://www.crd.york.ac.uk/prospero/ accessed on 11 February 2024). This review was conducted following the Preferred Reporting Items for Systematic Reviews and Meta-Analysis (PRISMA) guidelines [32]. A completed PRISMA checklist is provided in the Supplementary Materials.

### 2.1. Study Question

The main objective of this systematic review was to investigate the association between Hypothalamic-Pituitary-Adrenal (HPA) and Hypothalamic-Pituitary-Gonadal (HPG) axis-dependent hormones and bullying behavior in children and adolescents based on previous evidence. The research question was developed around the study’s main objective: Is there any relationship between HPA and HPG axis-dependent hormones and bullying behavior in 6–18-year-old children and adolescents?

### 2.2. Search Strategy

Two reviewers (IB and AA) conducted the literature search in four electronic databases: PubMed, Psycinfo, Scopus, and Web of Science between April 2023 and July 2023.

Different terms were used related to exposure (hormone, testosterone, estradiol, cortisol, dehydroepiandrosterone, HPA axis, HPG axis, 2D:4D ratio), outcome (bullying, peer victimization, peer aggression, school violence), and population (child, adolescent). After formulating the search strategy, it was adapted for each database. Subsequently, language (English and Spanish) and source-type (journal article) restrictions were applied.

As an example, the complete research string used for PubMed was: ((“hormon*”[Title/Abstract] OR “testosterone”[Title/Abstract] OR “estradiol”[Title/Abstract] OR “cortisol”[Title/Abstract] OR “dhea”[Title/Abstract] OR “dehydroepiandrosterone”[MeSH Terms] OR “hpa”[Title/Abstract] OR “hpg”[Title/Abstract] OR “2d:4d ratio”[Title/Abstract]) AND (“bullying”[MeSH Terms] OR “bully*”[Title/Abstract] OR “peer victimization”[Title/Abstract] OR “school violence”[Title/Abstract] OR “peer aggression”[Title/Abstract]) AND (“child*”[Title/Abstract] OR “child”[MeSH Terms] OR “adolescent*”[Title/Abstract] OR “adolescent”[MeSH Terms] OR “adolescent”[MeSH Terms] NOT “adult”[MeSH Terms] OR “adult”[Title/Abstract])).

### 2.3. Eligibility Criteria

The eligibility criteria are described in Table 1.

### 2.4. Study Selection and Data Extraction

To ensure consistency, two reviewers (IB and AA) defined the criteria and underwent training before beginning the search, screening, and data extraction process. The search yielded 152 results, out of which 88 were duplicated. Based on the eligibility criteria, the two reviewers independently screened titles, abstracts, and full texts. After the screening process, 33 articles were included in the study. Any disagreement between reviewers was solved by consensus discussion with a third expert (JI).

The data from selected articles were extracted independently by IB and AA, and then confirmed by a third researcher (NL). The following data were extracted from the articles: (1) study characteristics (author, year, design), (2) population characteristics (geographical location, sample size, age at the assessment of the exposure and outcome), (3) information about hormonal biomarkers (samples used and analysis method), (4) information about bullying assessment (instrument or scale used), (5) information about other variables of interest in the study (mediators, moderators, other variables), (6) main results of the study, and (7) information for assessing research quality.

### 2.5. Quality of Studies (Risk of Bias)

To assess the quality of each study, the two reviewers used the Robins E-tool, which is designed to assess observational epidemiologic studies mainly in the context of systematic reviews [33]. The tool assesses the quality of the studies on 7 domains including confounding, selection of participants in the study, classification of exposures, departures from intended exposures, missing data, measurement of outcomes, and selection of the reported result. As confounding factors, gender and age were considered, which are related to hormone levels and bullying. Based on the score, the risk of bias in the studies can be classified as low risk of bias, some concern about bias, high risk of bias, or very high risk of bias. High risk rates indicate that the study has serious methodological errors in analyzed domains.

## 3. Results

### 3.1. Literature Search and Study Selection

**Figure 1:**
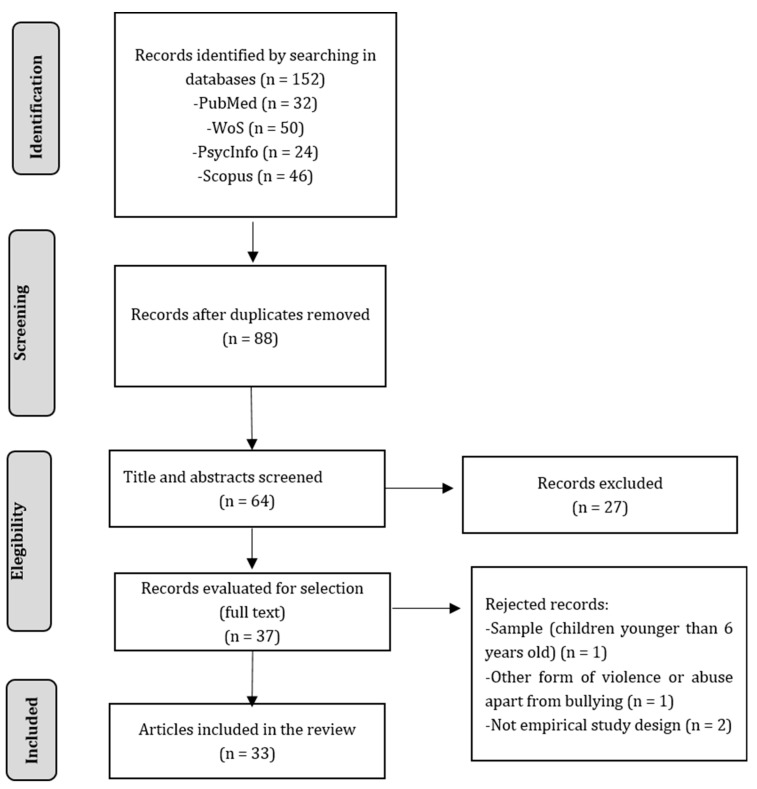
Flowchart of the systematic review.

The search initially yielded 152 records, but after removing duplicates, 64 records remained. Upon screening the titles and abstracts, 37 papers were identified for full text review. After a detailed reading of these articles, four of them were excluded based on the inclusion and exclusion criteria. Finally, a total of 33 studies were included in this systematic review. The process is summarized in Figure 1.

### 3.2. Description of Studies Included

Table 2 presents the general characteristics of the participants in the studies. The publication dates ranged from 2006 to 2023, with over a third (35%; *n* = 12) of the studies published during the last five years. One third of the studies were conducted in Europe (*n* = 11; 33%), another third was published in the USA (*n* = 11; 33%), eight in Canada (*n* = 8; 24%), two in China (*n* = 2; 6%), and one in Brazil (*n* = 1; 3%). Regarding study design, 18 were cross-sectional (*n* = 18; 55%) and 15 were longitudinal (*n* = 16; 45%). The studies used a variety of sample sizes, ranging from 31 participants [34] to 659 participants [35].

#### 3.2.1. Bullying Assessment

Bullying was assessed using various methods in the reviewed papers. A single study used peer nomination to identify the children and adolescents involved in bullying situations [53], while three relied on interviews conducted with participants’ mothers [48,49,64]. However, the majority of studies used self-reported questionnaires to identify participants´ bullying involvement. Although there is no single questionnaire used to assess bullying behavior, the most commonly used scales in the reviewed papers were “The Social Experiences Questionnaire” [38,44,58,61,62] and questionnaires based on the “Olweus Bully Victim Questionnaire” [29,31,35,54,59,65].

#### 3.2.2. Hormones Assessment

This study aimed to analyze the association between bullying behavior and HPA and HPG axis-dependent hormone levels. All but one study explored the role of cortisol in bullying. Most studies determined cortisol levels in saliva, except for four studies that determined cortisol levels in hair [35,36,51,52]. Saliva samples (as well as blood samples) refer to a time point cortisol levels or acute stress, whereas hair samples are used to collect information about cumulative cortisol levels and are used as an indicator of chronic stress levels.

With regard to saliva samples, cortisol reactivity, the cortisol-awakening response (CAR), diurnal cortisol curves, or total cortisol levels were studied, depending on the time of collection and the number of samples. To understand this, it is essential to know that cortisol levels follow a daily cycle that maintains healthy physiological functioning. Cortisol levels sharply increase in the morning, approximately 30 min after waking (known as the cortisol-awakening response). Subsequently, cortisol levels decline throughout the day, reaching their lowest points after sleep begins. Some articles rely on multiple saliva cortisol samples to study the cortisol secreted during specific hours by means of the cortisol curve or by measuring the area under the curve. Furthermore, as long as the HPA axis functions properly, the stress response and cortisol secretion follow predictable patterns and react by activating the HPA axis to stressful situations. However, this can change under chronic stress conditions.

Once the samples were collected, different analysis techniques were used to determine cortisol levels. More than 60% (*n* = 18) of the articles used enzyme immunoassays as hormone analysis techniques. In addition to this technique, studies analyzed hormones using several other techniques, such as luminescence or competitive radioimmunoassays.

Only three studies [29,30,31] have analyzed the role of HPG axis-dependent hormones in bullying behavior. All three studies measured testosterone levels in saliva, and one of them measured the participants’ 2D:4D ratio as an indicator of prenatal sex hormones [29].

### 3.3. Risk of Bias in Individual Studies

In our assessment, two studies were rated with some concerns of risk of bias (*n* = 2; 6%), three studies were deemed to have a very high risk of bias (*n* = 3; 9%), while the remaining (*n* = 28; 85%) were rated as having a low risk of bias. The overall risk of bias ratings has been detailed in Table 2, and we have summarized the rating for individual studies across seven domains: confounding, selection of participants into the study, classification of exposures, departures from intended exposures, missing data, measurement of outcomes and selection of the reported result measurement exposure (Appendix A).

Measurement of the exposure was the domain with a higher risk of bias rating since the methods used to measure exposure bullying were not validated and used only a few questions to identify participants as having bullying involvement. As a result, the rest of the domains were not evaluated, which was considered a very high bias risk. Those articles with “some concerns” are due to the selection of participants, since their characteristics may have affected the results.

### 3.4. Association between Bullying and Hormones

The association between bullying and hormone levels can occur in several ways. Previous evidence showed that hormones could be integrated in the study of bullying following two mechanisms. On the one hand, hormones could act as predictors of bullying. The dual hormone hypothesis states that high testosterone levels influence behaviors where social dominance is involved, as could be the case with bullying, and that elevated testosterone levels predict such behaviors when cortisol levels are low [19]. On the other hand, bullying has been identified as a stressful situation [2] and therefore it is understood that, as with other stressful events, it could alter the activity of the HPA axis.

To better understand the results, a distinction was made in depending not only on the hormones analyzed but also on the role (outcome, moderator, or predictor) played in this association between bullying and hormones.

#### 3.4.1. HPA-Dependent Hormones and Bullying

A single study focused on exploring the predictive role of cortisol and testosterone in bullying behavior [29]. Another ten studies explored the mediating role of cortisol (*n* = 10; 30%) [30,34,38,56,57,59,60,61,62]. The remainder (*n* = 21; 64%) studied the influence of bullying on cortisol levels.

*Cortisol as a predictor of bullying behavior*

In our previous study we analyzed the effects of testosterone and cortisol jointly with contextual factors on bullying behavior, exploring three different roles (victim, bully, bully/victim). Results showed that lower cortisol levels, together with a worse perceived school environment and less peer and social support, were associated with bullying involvement as a bully [29].

*Cortisol as an outcome of bullying behavior*

The association between cortisol levels and bullying behavior was examined in previous studies using different cortisol samples. When cortisol was analyzed in hair samples, mixed results were found. One study found that cumulative victimization was non-linearly related to hair cortisol levels, although peer victimization was not directly associated with HPA activity [52]. Another study showed that this association was nonlinear and sex-dependent. Boys who experienced moderate victimization had lower levels of hair cortisol, whereas boys who experienced higher victimization showed higher hair cortisol concentrations [51]. A third study concluded that, although polyvictimized (i.e., conventional crime, maltreatment, peer and sibling victimization, sexual victimization, and witnessing and indirect victimization) youth showed higher hair cortisol levels, peer and sibling victimization was not significantly related to hair cortisol levels [36]. Finally, in a previous study, we explored not only the associations between victimization by peers but also the implications participants have as bullies or bullies and victims. Results showed a trend association between being involved in bullying as a bully/victim and higher hair cortisol concentration (HCC). However, involvement as bullies or victims was not associated with higher HCC [35].

While some studies used hair samples, most of the analyzed studies used saliva samples. Regarding cortisol reactivity measures in saliva after a stressful situation, most studies concluded that victims showed lower cortisol reactivity than their counterparts [37,39,45,46,48,64]. Nevertheless, two studies found that bullied children and adolescents had elevated cortisol reactivity [41,43]. Finally, another two studies found no significant association between cortisol reactivity and peer victimization [44,63].

Other studies have explored the association between bullying behavior and total cortisol levels. Most of them found that bullying or peer rejection was associated with higher total cortisol levels [41,42,53]. Likewise, in 2017, Gonzalez-Cabrera et al. found that, in addition to cybervictims, cyberbullies/victims also had higher total cortisol levels [42]. However, another study did not observe a direct association between cortisol levels and victimization [40].

Moreover, some studies have analyzed the cortisol awakening response (CAR). According to two previous studies, bullied students had a lower cortisol awakening response [46,47], but other studies have not confirmed this association [38,42,59]. Most studies found that students who were bullied had flatter daily cortisol slopes [38,42,46,53]. However, two studies found no relationship between victimization and cortisol slopes or patterns [34,55,59].

Finally, according to some researchers, the relationship between victimization and cortisol levels may be sex-dependent. One studyfound that verbally bullied girls had lower cortisol levels than boys [54]. Additionally, some years later, another study concluded that bullied students had lower cortisol levels and flattened cortisol responses, but these associations were only statistically significant for boys [47].

*Cortisol as a moderator in the association of bullying and other mental health outcomes*

As mentioned above, seven of the studies reviewed examined the moderating or mediating role of cortisol levels (*n* = 7; 21%). The vast majority of the studies have focused on exploring the moderating role of cortisol levels in the association between bullying victimization and other psychological or mental health problems.

Three studies explored the moderating role of cortisol in the association between bullying victimization and depressive symptoms. These studies found that higher cortisol levels increase the association between peer victimization and depressive symptoms [56,58,60]. Moreover, three other studies have explored the moderating role of cortisol in the association between bullying victimization and aggression. One study showed that increased cortisol levels (AUC) buffered the link between victimization and next-day aggression only in boys [57]. Another study concluded that both, adolescents with high testosterone and high cortisol levels, and those with low testosterone and low cortisol levels responded more aggressively when victimized by peers [30]. Finally, one study observed that, at high levels of victimization, children with high cortisol levels presented not only higher levels of aggressiveness, but also higher levels of frustration. When victimization levels were low, children with lower cortisol levels also presented with higher levels of frustration.

In addition, a recent study found that blunted cortisol reactivity accounted for some of the effects of relational victimization on externalizing and internalizing problems, but this was only observed in boys [63]. Finally, one study analyzed the moderating role of cortisol in the effect of victimization on structural brain changes. They found that, in boys with a low daily cortisol output (assessed as the area under the curve [AUC]), high victimization was associated with a smaller right vlPFC (Ventrolateral Prefrontal Cortex) surface area. However, in the case of boys with a high AUC, high victimization was associated with a larger right vlPFC surface area. In addition, in boys with a steeper diurnal slope, it was concluded that high victimization was associated with a smaller right vlPFC surface area, whereas boys with a flatter diurnal slope showed that high victimization was associated with a larger right vlPFC surface area [59].

#### 3.4.2. HPG-Dependent Hormones and Bullying

Only three studies have investigated the association between HPG-axis-dependent hormone levels and bullying. First, in 2009 Vaillancourt et al. explored whether testosterone influenced peer victimization on bullying behavior. They concluded that the association between the two variables varied according to sex. Specifically, girls who were verbally bullied had lower testosterone levels than their counterparts, whereas among boys, those who were verbally bullied showed higher testosterone levels [31]. Testosterone levels have also been analyzed as a predictor of bullying through the analysis of the 2D:4D ratio, an indicator of prenatal sex hormone exposure. In this case, however, none of the studied variables were related to bullying behavior [29]. Finally, a recent study explored the moderating effects of testosterone and cortisol on the association between bullying and aggressive behavior. Results showed that adolescents with high testosterone and cortisol levels or with low testosterone and cortisol levels responded more aggressively when victimized or provoked [30].

## 4. Discussion

The main objective of this systematic review was to summarize the observational evidence assessing the association between HPA and HPG hormones and bullying behavior. Based on our selection criteria, 33 studies were included in the systematic review. To facilitate the interpretation of how these hormones and bullying behavior are related, we discuss the main findings separately for HPA hormones and HPG hormones, respectively.

Concerning the association between cortisol and bullying, a single study explored this relationship with cortisol as a predictor [29]. Seven studies examined the moderating role of cortisol in the association between bullying and other mental or psychological disorders and all found it to play an important role [30,56,57,58,59,60,63]. Finally, the association between cortisol levels and bullying behavior was examined using different cortisol samples analyzed in saliva and hair samples, and mixed results were found, as was concluded in a systematic review carried out in 2019 whichexplored the association between bullying behavior and cortisol levels determined using saliva [20]. Specifically, the HPA is both hyper-and hypo-responsive to social stressors. The reason for these confounding responses may depend not only on methodological concerns but also on the type of stressor and the time of occurrence. The HPA axis exhibits hyperactivated responses when the stressor is recent. Stress, however, can be detrimental as well when the stressor occurs distantly in time or is absent. These responses are consistent with the chronic stress hypothesis, which argues that the axis is activated when the stressor is initiated and that its continued activity causes a decrease in cortisol release below the normal levels and suggests dysregulation on the HPA axis [66].

Moreover, those that analyzed the effect of bullying behavior on chronic stress, using hair samples, also found mixed results. One recent systematic review concluded that the evidence exploring the associations between indicators of social adversity and hair cortisol in children provides inconsistent and limited results. They posit that this may be due to the existence of possible factors moderating the associations between adversity and physiological stress, such as the relationship with their caregiver, regulatory physiological processes, genetic factors that could influence the perception or response of stress, or environmental factors affecting hair cortisol levels [67].

Regarding the association between HPG hormones and bullying behavior, only one previous study that explored the association between prenatal androgen levels and bullying behavior found no statistically significant result. The main reason why this study did not find a correlation between aggressive behavior and the 2D:4D index, may be the sample size [29]. Additionally, in contrast to previous studies, the participants in this paper were younger than participants in other studies, which established an association between prenatal androgen levels and aggressive behavior. Among HPG hormone levels, pubertal testosterone was studied in relation to bullying involvement, specifically in three studies. One found no association between testosterone and bullying behavior [29]. The second concluded that the association between the two variables varied according to sex. Girls who were verbally bullied had lower testosterone levels than their counterparts, whereas boys who were verbally bullied showed higher testosterone levels [31]. Finally, Calvete and Orue in their recent study showed that adolescents with high testosterone and cortisol levels or with low testosterone and cortisol levels responded more aggressively when victimized or provoked [30]. The reason why one of the studies did not find a statistically significant association between the variables may be because the participants in this study were younger than the participants in the other two. As testosterone levels increase at puberty and participants in our study were in prepubertal stages, their testosterone levels may be very low and homogeneous.

### Strengths and Limitations

To the best of our knowledge, this is the first systematic review analyzing jointly the association that HPG- and HPA-dependent hormones have with bullying behavior. We conducted this review following the PRISMA statement guidelines and documented the methods in a protocol registered on PROSPERO before starting the review, strengthening the trustworthiness of the process and results. Additionally, for the search, no limits were placed on publication dates and study types. However, several limitations should be noted too. First, we limited to studies written in either English or Spanish. Furthermore, reviewers were not blinded to the study authors and affiliations during the process.

Regarding the studies, there was considerable methodological heterogeneity between studies; and the use of different instruments to measure bullying or different methods to determine hormone levels may have contributed to these mixed results. In addition, few studies have explored the association of the different roles of bullying behavior.

## 5. Conclusions

The studies analyzed in our review in general were of high quality, but some gaps have been identified. For future research, as noted in the method section, we recommend exploring the effect of different hormones of the HPA and HPG axes and using a broader set of biomarkers (i.e., estradiol, dehydroepiandrosterone, LH, or FSH hormones). This recommendation would respond to the fact that these axes have been shown to be linked in the previous literature [19,66].

Additionally, certain methodological aspects should also be taken into account when evaluating these hormones. The HPA and HPG axes are not static, and their activity varies at different stages of development. Specifically, during puberty, they undergo an increase in their activity, so that assessing the pubertal stage and controlling for this would be the most appropriate methodologically. Likewise, in terms of bullying assessments, it has also been found that various methods are used, such as questionnaires, interviews, or peer nominations. It would be highly recommended for future studies the use previously validated scales or questionnaires (i.e., OBVQ). Finally, future studies should investigate the relationship between cyberbullying and hormone levels, as there is scarce evidence of this association. Further research is needed to better understand how bullying affects physical and emotional development during childhood and adolescence. Biological measures may not only improve our understanding of aggressive behavior but also guide the development of prevention and management programs for it.

## Figures and Tables

**Table 1:** Inclusion and exclusion criteria.

Inclusion Criteria	Exclusion Criteria
School aged children (6–12 years) or adolescents (12–18 years) at the age of the assessment of the outcome.	Preschool children (0–6 years) Adults (>18 years)
HPA and HPG axis-dependent hormones.	Other hormones Other biomarkers
School bullying behavior (traditional or cyberbullying)	Other maltreatment or aggressive behavior

**Table 2:** Main characteristics of the included studies.

Author and Year	Study Design	Country, Sample Size (N), Age	Hormonal Marker	Bullying Assessment	Other Variables	Main Findings	Overall Risk of Bias
Relation between Testosterone and Bullying							
Vaillancourt et al. (2009) [31]	Cross-sectional	Canada, N = 151, M [SD] = 12 years and 7 months [0.76 for boys and 0.72 for girls]	Salivary testosterone (two samples). Analysis technique: enzyme immunoassay	Peer victimization: Empirically validated self-report questionnaire adapted from OBVQ	Pubertal Development Scale (PDS), age, time and day of sampling	Verbally bullied girls had lower testosterone levels than their non-bullied peers Verbally bullied boys had higher testosterone than their non-bullied peers	Low Risk of Bias
Babarro et al. (2022a) [29]	Cross-sectional	Spain, N = 302, 11-year-old	2D:4D ratio, salivary cortisol and testosterone (two saliva samples). Analysis technique: Enzyme immunoassay kit	Bullying: Short version of Olweus Bully Victim Questionnaire (OBVQ)	Structural Equation Modeling (SEM) analysis, other variables: Risky decision maker, quality of family interactions, social context	Lower salivary cortisol levels were associated with bullying involvement as a bully	Low Risk of Bias
Calvete et al. (2023) [30]	Cross-sectional	Spain, N = 577, M [SD] = 14.64 [0.96]	Salivary cortisol and testosterone (one sample). Analysis technique: electrochemiluminescence Immunoassay	Victimization: The Revised Peer Experiences Questionnaire Cyberbullying: The Cyberbullying Questionnaire	Sex	When victimized or provoked by peers, a more aggressive behavior was shown by adolescents with high testosterone and high cortisol or low testosterone and low cortisol The testosterone/cortisol ratio was associated with aggressive behavior only in case of girls	Low Risk of Bias
Cortisol is outcome							
Araújo de Azeredo et al. (2020) [36]	Cross-sectional	Southern Brazil, N = 83, M [SD] = 10.84 [1.36]	Hair cortisol concentration (30 days). Analysis technique: enzyme-linked immunosorbent assay (ELISA)	Lifetime victimization experiences: Portuguese version of the Juvenile Victimization Questionnaire—2nd revision (JVQ-R2)	Gender, age, socioeconomic status, and mental health problems (internalizing and externalizing symptoms, Child Behavior Checklist (CBCL))	Youths who reported high levels of polyvictimization had higher hair cortisol concentration compared to youths exposed to less victimization No differences were observed in the subdomain peer and sibling victimization alone	Some Concerns
Babarro et al. (2022b) [35]	Cross-sectional	Spain, N= 659, M [SD] = 10.95 [0.46]	Hair cortisol concentration (3 months). Analysis technique: competitive radioimmunoassay (RIA).	Bullying: short version of OBVQ	SEM analysis, other variables: School environment, problems with peers, executive function (risky decision making)	Being involved as a bully/victim was related to higher Hair Cortisol Concentration (HCC) (trend association), but being involved as a bully or a victim was not related to HCC	Low Risk of Bias
Bendezú et al. (2022) [37]	Cross-sectional	USA, N = 157 girls, M [SD] = 14.72 [1.38]	Salivary cortisol reactivity before and after TSST (Trier Social Stress Test, four samples). Analysis technique: high sensitivity enzyme immunoassay.	Peer victimization: Revised Peer Experiences Questionnaire (RPEQ)	Pro-inflammatory cytokines in saliva, PDS, chronic peer strain, depressive symptoms, Body Mass Index (BMI), caregiver education, family-related stress…	Adolescents with low cortisol response and stably low cytokine levels experienced lower levels of peer stress exposure Low cortisol response and stably high cytokine adolescents experienced greater peer stress exposure	Low Risk of Bias
Brendgen et al. (2017a) [38]	Longitudinal	Canada, N = 272 (136 MZ twin pairs) M [SD] = 14.07 [0.76 for boys and 0.72 for girls]	Salivary cortisol (four consecutive days, four samples each day). Analysis technique: enzyme immunoassay	Peer victimization: Social Experiences Questionnaire	Depression symptoms, physical health, pubertal status	Twin differences in peer victimization and a problematic mother–child relationship was significantly related to twin differences in diurnal cortisol secretion after controlling for potential confounders	Low Risk of Bias
Calhoun et al. (2014) [39]	Cross-sectional	USA, N = 62, M [SD] = 14.70 [1.33])	Salivary cortisol pre and after a Social Stressor Task (three samples). Analysis technique: high-sensitive enzyme immunoassay	Peer victimization: subscale of the Revised Peer Experiences Questionnaire	Friendship quality, cortisol timing, pubertal stage, depressive symptoms, life events	Higher levels of relational victimization were associated with blunted HPA reactivity High-quality friendship was associated with a better HPA axis recovery	Some Concerns
Carney et al. (2010) [40]	Cross-sectional	USA, N = 91, M = 11.5	Salivary cortisol (two samples). Analysis technique: enzyme immunoassay	Bullying: Exposure to Bullying Events	Anxiety	Greater exposure to bullying jointly with general anxiety was associated with lower cortisol levels An indirect effect of EBE was observed on cortisol levels through general anxiety	Very High Risk of Bias
Chen et al. (2018) [41]	Cross-sectional	China, N = 80, M [SD] = 10.83 [0.7]	Salivary cortisol reactivity to TSST (six samples). Analysis technique: ELISA	Bullying: OBVQ	None	Children with a history of victimization had higher cortisol levels (cortisol reactivity and total cortisol concentration) in comparison to those without a history of bullying	Low Risk of Bias
González-Cabrera et al. (2017) [42]	Longitudinal	Spain, N = 60, M [SD] = 15.58 [1.12]	Salivary cortisol (five samples). Analysis technique: electrochemiluminescence	Peer cyberbullying: questionnaire that consists of 45 items	Perceived stress, anxiety	Cortisol secretion varied depending on the role of adolescents in cyberbullying: cybervictims and cyberbully-victims exhibited higher cortisol secretion as compared to cyberbullies and cyberbystanders Relationships between cyberbullying victimization at Wave 1 and anxiety and perceived stress at Wave 2 are explained by higher AUC values	Low Risk of Bias
Kliewer et al. (2006) [43]	Cross-sectional	USA, N = 101 African-American youth, M [SD] = 11.14 [1.28]	Salivary cortisol before and after a laboratory task (three samples). Analysis technique: enzyme immunoassay	Peer victimization: eight items from Ewart’s Adolescent Resource Challenges Scale	Witnessed violence, age, gender, internalizing symptoms, major life events	Peer victimization was related to lower basal cortisol levels Victimization was associated with an increase in cortisol secretion	Low Risk of Bias
Kliewer et al. (2012) [44]	Longitudinal	Country: NA, N = 228 (45% male, 90% African American), M [SD] = 14 [1.6]	Salivary cortisol (four samples). Analysis technique: enzyme immunoassay	Peer victimization: The Social Experience Questionnaire	Aggression, time of day, pubertal status, medication use	Non-significant associations between victimization/aggression and salivary cortisol Aggression moderates the relationship between relational peer victimization and physiological responses to stress	Low Risk of Bias
Kliewer et al. (2016) [45]	Longitudinal	USA, N = 242, M [SD] = 11.98 [1.56]	Salivary cortisol response after stress interview (6 samples). Analysis technique: enzyme immunoassay	Peer victimization: Survey of Children’s Exposure to Violence	Emotion regulation, age, sex, interview start time, pubertal status	Victimization was negatively related to total cortisol output (Area Under the Curve (AUC)) Emotion regulation had a protective effect on the relationship between victimization and cortisol	Low Risk of Bias
Knack et al. (2011) [46]	Longitudinal	USA, N = 107 (56.1% girls), M [SD]= 12.23 [1.09]	Salivary cortisol (four saliva samples during two consecutive school days, in two phases). Analysis technique: enzyme immunoassay	Peer victimization: Children’s self-experiences questionnaire	Physical health	Victimized adolescents had lower cortisol levels at 30 min after waking and 30 min before bed During the TSST, victims reported more stress and altered cortisol reactivity CAR mediated the link between victimization and health problems	Low Risk of Bias
Östberg et al. (2018) [47]	Cross-sectional	Sweden, N= 392 (cortisol subsample n = 198), 14–16 years-old	Salivary cortisol (four samples). Analysis technique: competitive radioimmunoassay (RIA)	Bullying: identified through the question: “Sometimes troublesome things happen at school. How often do the following things happen to you at school?”	Stress, recurrent pain	Bullied students had lower total cortisol (Area Under the Curve: AUC) and lower cortisol awakening response (Cortisol Awakening Response: CAR) compared to those who were not bullied	Very High Risk of Bias
Ouellet-Morin et al. (2011a) [48]	Longitudinal	Great Britain, N = 60, M [SD] = 12.53 [0.52]	Salivary cortisol reactivity to Psychosocial Stress Test (PST; five samples). Analysis technique: Immunoassay.	Bullying victimization: interviews	Child-specific family environments, concomitant, stress-related individual factors	Bullied and non-bullied monozygotic (MZ) twins showed distinct patterns of cortisol secretion after the PST. Bullied twins showing a blunted cortisol response	Low Risk of Bias
Ouellet-Morin et al. (2011b) [49]	Longitudinal	Great Britain, N= 190, 12-year-old children	Salivary cortisol reactivity to PST (five samples). Analysis technique: Immunoassay	Bullying victimization: interviews	Social, emotional, and behavioral problems	Maltreated/bullied children showed lower HPA axis reactivity to stress	Low Risk of Bias
Ouellet-Morin et al. (2013) [50]¦	Longitudinal	Great Britain, N = 56 (28 pairs) twins), 12-year-old	Salivary cortisol reactivity to PST (five samples). Analysis technique: Immunoassay	Bullying victimization: interviews	DNA methylation analysis	Bullied and non-bullied twins showed distinct patterns of cortisol response. Bullied twins did not exhibit the expected cortisol increase after the PST	Low Risk of Bias
Ouellet-Morin et al. (2021a) [51]	Longitudinal	Canada, N= 556, 17-year-old	Hair cortisol concentration (3 months). Analysis technique: luminescence immunoassay	Peer victimization: Self-Report Victimization Scale	Depressive, medications, sleeping habits, BMI, tobacco, drug and alcohol consumption, socioeconomic status (SES), single parenthood, mothers’ and fathers’ education and occupational prestige, behavioral problems	The association between peer victimization and hair cortisol was non-linear in boys: those who experienced moderated peer victimization had lower HCC, but higher levels of victimization were related to higher HCC.	Low Risk of Bias
Ouellet-Morin et al. (2021b) [52]	Longitudinal	Canada, N= 556, 17-year-old	Hair cortisol concentration (3 months). Analysis technique: luminescence immunoassay	Peer victimization: Self-Report Victimization Scale	Other indicators of adversity (young motherhood, single-headed family, SES, maternal alcohol use, hostile-reactive parenting, maternal depressive symptoms, neighborhood dangerousness)	The association between chronic adversity and HCC was non-linear: Those adolescents with lower and higher levels of adversity had moderate-to-higher HCC, compared with participants with moderate levels of adversity compared that had lower HCC Peer victimization taken separately was not associated with HCC	Low Risk of Bias
Peters et al. (2011) [53]	Cross-sectional	Netherlands, N = 97, M [SD] = 9.27 [0.2]	Salivary cortisol (2 consecutive days, five samples each day). Analysis technique: time-resolved fluorescence immunoassay	Peer victimization: Peer nominations	Number of friends, friendship quality, behavior problems, gender	Those children who were excluded from peer groups showed elevated cortisol levels at school and flattered diurnal cortisol curves Peer victimization was not directly associated with HPA activity	Low Risk of Bias
Vaillancourt et al. (2008) [54]	Cross-sectional	Canada, 154, M [SD] = 147 [9.07] months	Salivary cortisol (three days, two samples each day). Analysis technique: enzyme immunoassay	Peer victimization: OBVQ	Sex, pubertal status, age, depression, anxiety	For boys, occasional exposure was associated with higher cortisol levels For girls, exposure was associated with lower cortisol levels	Low Risk of Bias
Vaillancourt et al. (2011) [55]	Longitudinal	Canada, N = 168 (91 boys), M [SD] = 147 [9] months	Salivary cortisol (two days, two samples each day). Analysis technique: enzyme immunoassay	Peer victimization: OBVQ	Depressive symptoms, memory	Peer victimization at T1 predicted elevated depressive symptoms in T2 and at the same time, depressive symptoms in T2 predicted lower salivary cortisol in T3	Low Risk of Bias
Williams et al. (2017) [34]	Cross-sectional	USA, N = 31, 14–16 (M = 14.5)	Salivary cortisol (two samples). Analysis technique: NA	Bullying: Personal Experiences Checklist (PECK)	Depression, PDS	No statistically significant correlations were found between cortisol and bullying	Low Risk of Bias
Cortisol is mediator							
Adams et al. (2021) [56]	Cross-sectional	Canada, N= 113, from grade 5 (M = 10.31 and grade 6 (M = 11.33).	Salivary cortisol (four days, five samples each day). Analysis technique: DSL kit NA	Peer victimization: three items adapted from Hamburger et al. (2011).	Depression, three items adapted from Child Depression Inventory	Peer victimization was indirectly related to depressive symptoms via cortisol, but only at high rates of chronic victimization	Very High Risk of Bias
Arbel et al. (2019) [57]	Longitudinal	Country: NA, N = 99, M [SD]= 18.06 [1.09]	Salivary cortisol (three consecutive days, five samples each day). Analysis technique: enzyme immunoassay	Peer victimization: adapted from the How Friends Treat Each Other scale.	Age, cotinine levels, hours of sleep, time of morning awakening, use of medications	In boys, the association between victimization and next-day perpetration was buffered by increases in AUG	Low Risk of Bias
Brendgen et al. (2017b) [58]	Cross-sectional	Canada. N= 406 (203 twin pairs), M [SD]= 14.07 [0.3]	Salivary cortisol (4 consecutive days, one sample each day). Analysis technique: high sensitivity enzyme immunoassay	Peer victimization: Social Experiences Questionnaire.	Depression	There was no genetic association between depression symptoms and peer victimization in individuals with low or moderate levels of cortisol secretion, but a genetic association emerged in those with high levels Cortisol levels in the morning were associated with depression symptoms as peer victimization increased	Low Risk of Bias
Du Pleiss et al. (2019) [59]	Longitudinal (Association between victimization and cortisol cross-sectional)	Netherlands, N = 50, M [SD] = 9.29 [0.37]	Salivary cortisol (two consecutive days, five samples each day). Analysis technique: time-resolved fluorescence immunoassay (DELFIA)	Victimization: OBVQ	Neuroimaging	Cortisol moderated the relationship between childhood victimization and adolescent vlPFC structure in boys Victimization and cortisol showed no significant associations	Low Risk of Bias
Iob et al. (2021) [60]	Longitudinal	Great Britain, N = 300 (150 twin pairs), 11-year-old	Salivary cortisol (pre- and post-task). Analysis technique: high sensitivity chemiluminescence assay	Bullying: Adverse childhood experiences (ACE)	Depressive symptoms, latent genetic risk scores, sex, socioeconomic status	Children exposed to three or more ACEs had lower cortisol levels at age 11 and elevated depressive symptoms at age 21 The mediation analysis indicated that cortisol mediated associations of ACEs cumulative exposure, bullying, and dysfunctional parenting/emotional abuse with depressive symptoms.	Low Risk of Bias
Rudolph et al. (2010) [61]	Cross-sectional	Country: NA, 132 children, M [SD]= 9.46 [0.33]	Salivary cortisol (three samples). Analysis technique: highly sensitive enzyme immunoassay	Peer victimization: Social Experiences Questionnaire	Aggression, frustration	When victimization levels were high, children with heightened cortisol levels had greater frustration than children with dampened cortisol levels At low levels of victimization, children with dampened cortisol levels had higher frustration scores compared to those with heightened cortisol levels At high levels of victimization, children with heightened cortisol levels had aggression scores greater than children with dampened cortisol levels	Low Risk of Bias
Rudolph et al. (2011) [62]	Longitudinal	USA, 132 children, M [SD] = 9.46 [0.33]	Salivary cortisol (three samples). Analysis technique: highly sensitive enzyme immunoassay	Peer victimization: Social Experiences Questionnaire	Depressive symptoms, rumination, medication usage	At high levels of victimization, children with heightened anticipatory cortisol had greater depressive symptoms than children with dampened anticipatory cortisol At low levels of victimization, children with dampened anticipatory cortisol had greater depressive symptoms than children with heightened anticipatory cortisol	Low Risk of Bias
Sun et al. (2022) [63]	Cross-sectional	China, N = 150, M [SD]= 10.69 [0.93]	Salivary cortisol reactivity after PST (six samples). Analysis technique: Immunosorbent assay.	Peer victimization: Multidimensional Peer Victimization Scale.	Internalizing and externalizing problems, gender, age, BMI, socioeconomic status	Blunted cortisol reactivity explained part of the effect of relational victimization on internalizing and externalizing problems, only for boys	Low Risk of Bias

Note: M = Mean; SD = Standard Deviation; NA = No information Available.

**Figure A1:**
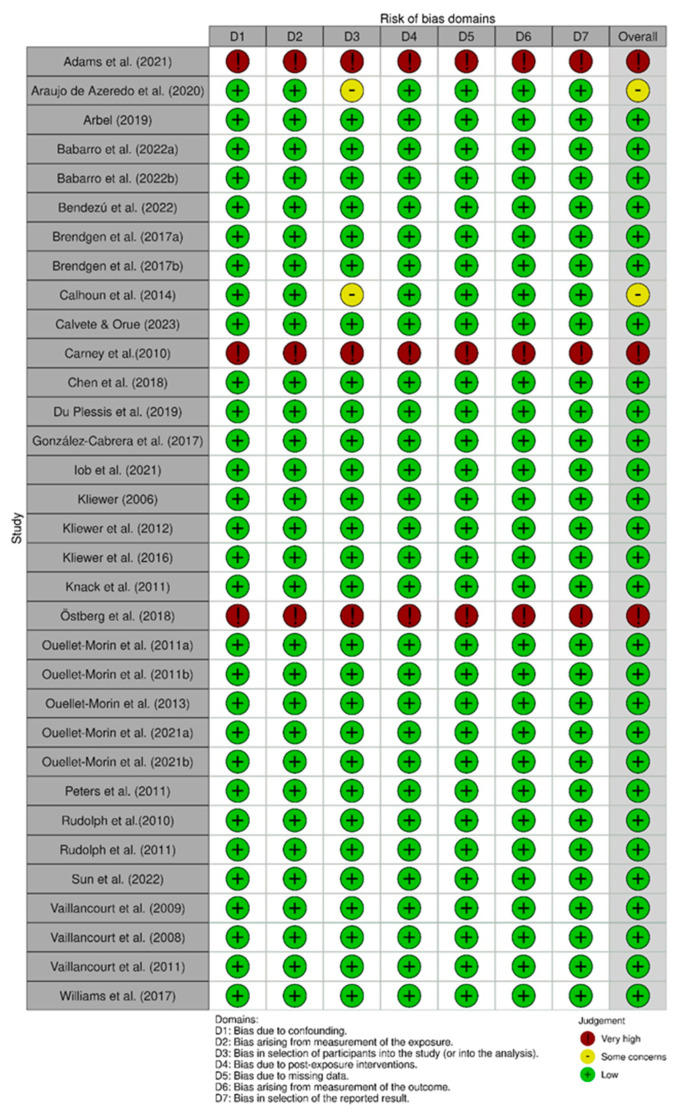
Traffic-light plot summarizing the risk of bias in the selected studies. Note: Studies analyzed [29,30,31,34,35,36,37,38,40,41,42,43,44,45,46,47,48,49,50,51,52,53,54,55,56,57,58,59,60,61,62,63,64,67].

## Data Availability

Not applicable.

## Supplementary Materials

The following supporting information can be downloaded at: https://www.mdpi.com/article/10.3390/children11020241/s1, Table S1: PRISMA 2020 Checklist.
